# The dynamic mask: Facial correlates of character portrayal in professional actors

**DOI:** 10.1177/17470218211047935

**Published:** 2021-10-04

**Authors:** Matthew Berry, Steven Brown

**Affiliations:** Department of Psychology, Neuroscience & Behaviour, McMaster University, Hamilton, Ontario, Canada

**Keywords:** Acting, characters, embodiment, emotions, facial expression, facial expansion, facial contraction, performance

## Abstract

Actors make modifications to their face, voice, and body to match standard gestural conceptions of the fictional characters they are portraying during stage performances. However, the gestural manifestations of acting have not been quantified experimentally, least of all in group-level analyses. To quantify the facial correlates of character portrayal in professional actors for the first time, we had 24 actors portray a contrastive series of nine stock characters (e.g., king, bully, lover) that were organised according to a predictive scheme based on the two statistically independent personality dimensions of assertiveness (i.e., the tendency to satisfy personal concerns) and cooperativeness (i.e., the tendency to satisfy others’ concerns). We used three-dimensional motion capture to examine changes in facial dimensions, with an emphasis on the relative expansion/contraction of four facial segments related to the brow, eyebrows, lips, and jaw, respectively. The results demonstrated that expansions in both upper- and lower-facial segments were related to increases in the levels of character cooperativeness, but not assertiveness. These findings demonstrate that actors reliably manipulate their facial features in a contrastive manner to differentiate characters based on their underlying personality traits.

## Introduction

During theatrical performances on stage and screen, an actor has to create the physical portrayal of a fictional character, one who could reasonably exists within the scenario of the presented storyworld. An actor not only has to portray this person, but also has to persuade others that they are indeed this person, doing so using physical alterations to their face, voice, and body (Dusinberre, 1998; Smith, 1971). Many theatrical traditions cross-culturally have relied on the donning of masks by actors to convey a character’s prototypical facial expressions (Benedetti, 2012; Meineck, 2011; Storm, 2016). The nature of masks has evolved over the last two millennia from being the most static representations of expressions to being highly dynamic (Benedetti, 2012; Kristeller, 1951; Meineck, 2011; Mirodan, 2019). However, our interest in this study is to quantify the actual facial changes that actors generate when portraying contrastive characters, where these facial changes serve metaphorically as a type of “dynamic mask” for each character.

We do this in the context of emerging research programmes on the nature of acting that attempt to systematise the “gestural codes” used by actors to create portrayals of fictional characters for both academic and practical uses on stage (Berry & Brown, 2019; Kemp, 2012; Konijn, 2000). While acting theorists since the time of Aristotle have debated about whether actors have to actually feel the emotions that they are portraying on stage (Archer, 1888/2012; Aristotle, 335 BCE/1996; Diderot, 1830/2019; Stanislavski, 1936/1989), our focus will be on the external manifestations of such expressions, rather than on their internal driving forces. The challenge for an actor is the same regardless of the method they use to get into character: they have to produce a realistic and compelling representation of a person who they themselves are not, and have to persuade an audience that they are indeed the embodiment of this person (Weinbren, 2020).

While there has been a rich tradition of looking at the facial correlates of emotion—one that dates back to Darwin, if not before—there has not been a comparable research programme on the facial correlates of character. To the best of our knowledge, this study is the first one to quantify the facial correlates of character portrayal separate from emotions associated with characters. While a small number of studies have examined characters as vehicles for expression—for example, using cartoon characters (Liu, Chen, & Chang, 2019; Zhao, Meng, An, & Wang, 2019), virtual models (Hahn, Castillo, & Cunningham, 2019), avatars (Ennis, Hoyet, Egges, & McDonnell, 2013; Tinwell, Grimshaw, Nabi, & Williams, 2011; Wallraven, Bülthoff, Cunningham, Fischer, & Bartz, 2007), and robots (Lin et al., 2013)—the tasks analysed in these studies have been firmly rooted in measurements of emotion, rather than the characters themselves.

In like form, there has been a long practice in experimental studies of facial emotion of employing professional actors to create images of prototypical facial expressions so as to serve as stimuli for perceptual discrimination studies of emotional expression (Carroll & Russell, 1997; Ershadi, Goldstein, Pochedly, & Russell, 2018; Scherer & Ellgring, 2007a, 2007b; Wallbott & Scherer, 1986). However, there has been far less interest in looking at the production process itself and in understanding the inherent diversity of expressions for any given emotion across a group of actors (Mehu & Scherer, 2015; Scherer & Ellgring, 2007a, 2007b; Wallbott & Scherer, 1986), instead placing the focus on the highly discriminable prototypes that are used in perceptual experiments. (For studies examining actors’ portrayals of emotions, see the studies by Carroll & Russell, 1997; Ershadi et al., 2018; Scherer & Ellgring, 2007a, 2007b; Wallbott & Scherer, 1986.) Indeed, Scherer and Ellgring (2007a) remarked that Darwin (1872/1998) may have been the first and last scholar to independently address the underlying principles of facial configurations and to detail the conditions under which particular facial configurations are produced for specific emotions.

### Categorical versus dimensional models and measures of emotion

Two general approaches to the classification of emotions have looked at the relationships among them as being either categorical or dimensional, respectively. The validation of such approaches has been strongly grounded in the analysis of the facial correlates of the emotions. Categorical schemes of discrete emotions date back historically to Darwin (1872/1998), but were developed in great detail by more recent scholars such as Tomkins (1962, 1963, 1984) and most especially by Ekman and his colleagues (Barrett, Adolphs, Marsella, Martinez, & Pollak, 2019; Bartlett et al., 2005; Ekman, 1984, 1992, 1993; Ekman & Friesen, 1972, 1978a, 1978b; Ekman & Rosenberg, 1997, 2005; Izard, 1971, 1992, 1993a, 1993b; Rosenberg & Ekman, 2020; for a comparison between discrete and componential emotion theories, see the study by Scherer & Ellgring, 2007a). Basic emotion theory (BET; as coined by Russell, 2009) is the most popular categorical scheme of the emotions, positing that there is a small number of categorically distinct emotions, each of which addresses a distinct biological function (Frijda, 2007; Panksepp, 2010). Canonical basic emotions include anger (i.e., hot and cold), happiness (i.e., amusement, contentment, and satisfaction), disgust, embarrassment, excitement, fear, guilt, pride, relief, sadness, and shame (see Chapter 3 in the study by Dalgleish & Power, 1999).

Different numbers and combinations of basic emotions have been used to study actor-generated emotional displays for perceptual discrimination studies across multiple modalities, such as the voice (Scherer & Ellgring, 2007b; Scherer, Banse, Wallbott, & Goldbeck, 1991; Wallbott & Scherer, 1986), body (Scherer & Ellgring, 2007b; Wallbott, 1998; Wallbott & Scherer, 1986), and face (Carroll & Russell, 1997; Mehu & Scherer, 2015; Scherer & Ellgring, 2007a, 2007b). In such cases, distinct emotions are analysed categorically utilising modular schemes for the face. For instance, in the Facial Action Coding System (FACS) developed by Ekman and colleagues, facial movements are analysed with respect to action units (AUs), which are observable actions of the underlying facial musculature (Barrett et al., 2019; Bartlett et al., 2005; Ekman, 1984, 1992, 1993; Ekman & Friesen, 1972, 1978a, 1978b; Ekman & Rosenberg, 1997, 2005; Izard, 1971, 1992, 1993a, 1993b; Rosenberg & Ekman, 2020; Zhi, Liu, & Zhang, 2020). Such AUs can function either singularly or combinatorially.

Facial analyses from the BET perspective describe how prototypical facial expressions are the result of particular combinations of AUs. For example, Scherer and Ellgring’s (2007a) facial analysis of actors demonstrated that displays of happiness were accompanied by AU configurations involving smiles (i.e., cheeks raising, lip corners pulling out, and jaw lowering), whereas displays of panic or fear were accompanied by AU configurations resembling a scream (i.e., brow movement and jaw lowering). One critique of categorical schemes is that they lack any concept of emotional intensity, such as that between happiness and elation or between fear and terror (Scherer & Ellgring, 2007a). Another critique is that they offer complex descriptions of activation patterns that reduce accessibility for actors consulting scientific literature to improve performance techniques. Both of these critiques can be addressed through the adoption of more robust organisational schemes.

In contrast to categorical schemes, dimensional schemes classify the emotions along a number of continua. A prominent example is the “core affect” theory and its structural analogue—the circumplex model—which have been used to examine the distribution of emotions along the two emotion dimensions of valence (the pleasure–displeasure continuum) and arousal (the activation–deactivation continuum) (Posner, Russell, & Peterson, 2005; Russell, 1980, 1994, 2005, 2009; Russell & Bullock, 1985; Vesker, Bahn, Degé, Kauschke, & Schwarzer, 2018). For example, Mehu and Scherer (2015) conducted a joint production and perception study of professional actors, comparing categorical and dimensional models of emotion. Their results demonstrated that displays of positive-valence + high-arousal emotions correlated with raising of the brow, raising of the cheeks, parting of the lips, and lowering of the jaw. By contrast, negative-valence + high-arousal emotions were correlated with both raising and lowering of the brow (depending on the emotion), widening of the eyes, lowering of the lip and jaw, and pressing of the lips together. The authors pointed out that “ . . . rather than being specific to discrete emotions or emotional dimensions, facial behavior reflects combinations of the general dimensions underlying emotional experience, combinations that appear to cut across discrete emotion categories” (Mehu & Scherer, 2015, p. 807).

While categorical approaches to facial expression look at the face as a collection of individual muscle-related units, there is also the sense in which the face has larger functional units than that. Both intuition and neuroanatomical innervation patterns (Wilson-Pauwels, Akesson, Stewart, & Spacey, 2002) suggest that an expression like a smile is not just the activation of a collection of individual AUs, but that it is the expansion of a horizontal segment extending between the lateral edges of the lips, whereas a frown is a retraction of that same segment. Similarly, the expression of surprise is an expansion of a vertical segment extending between the upper and lower lips through a lowering of the jaw, whereas the expression of disgust is a retraction of that same segment.

A number of studies have applied segmental approaches to the analysis of facial expression, employing geometric or point-to-point distance-based features to analyse changes in segment length caused by the recruitment of the underlying musculature (Fasel & Luettin, 2003; Hammal, Couvreur, Caplier, & Rombaut, 2007; Hammal & Massot, 2010; Kanade, Cohn, & Tian, 2000; Livingstone, Thompson, Wanderley, & Palmer, 2015; Sandbach, Zafeiriou, Pantic, & Yin, 2012; Schmidt, Cohn, & Tian, 2003; Soyel & Demirel, 2007; Valstar, Gunes, & Pantic, 2007; Yacoob & Davis, 1996; Y. Zhang, L. Zhang, & Hossain, 2015). For example, Schmidt and colleagues (2003) measured participants’ smiles by analysing a segment extending from the centre of the lip to the lip corner, and obtained continuous data for its position, duration, amplitude, velocity, and acceleration. Soyel and Demirel (2007) attempted to classify seven basic emotions using distance vectors related, respectively, to eye opening, eyebrow raising, mouth opening vertically and horizontally, and lip stretching. The authors found that expansion and contraction of these five segments provided adequate information to automatically discriminate facial emotions with a mean accuracy of 91.3%, outperforming other two-dimensional (2D) and three-dimensional (3D) recognition systems in their analysis (Soyel & Demirel, 2007). Metrics such as segmental expansion and contraction are far more intuitive and transparent to understand than AUs for non-specialists, such as professional actors.

### Multimodal expression

Emotional expression is an intrinsically multimodal activity, producing correlated changes in the face, body, and voice. While certain types of actors do not speak while acting, most notably mime actors, the vast majority of actors do. Studies of dramatic acting during line recitation—i.e., a monologue—are, therefore, necessary to maximise the ecological validity of experimental studies. However, the production of emotional expression with the face during acting interacts with the articulatory movements of the lips, cheeks, and jaw during speaking. Such movements are necessary for the production of phonemes as well as for the production of facial movements that contribute to the vocal expression of emotion separate from articulation. Therefore, any experimental study of facial expression during dramatic acting needs to control for speech-related facial movements to disambiguate articulation effects from purposeful facial expression when delivering lines.

The majority of studies of emotional expression are unimodal. As Scherer and Ellgring (2007b) noted, multimodal analyses of emotional expression, specifically speech, are rare despite the clear integration of the face, voice, body, and postural communicative channels. Some multimodal studies of facial expression in the context of vocal production have focused on singing (Livingstone et al., 2015; Thompson & Russo, 2007), where correlations have been observed between vocal pitch and both raising of the brow and lowering of the jaw, while other studies have focused on speech (Livingstone et al., 2015; Scherer & Ellgring, 2007b). In their study of multimodal expression, Scherer and Ellgring (2007b) observed correlated changes between the voice and face for several basic emotions, including correlations of both high pitch and loudness with activations in AUs for the brow, cheek, and jaw. In this study, we take a multimodal approach to acting by examining facial expression in the context of speech production, and attempt to disambiguate movements of the lower face associated with emotional expression from those associated with speech articulation. We also look at correlations between facial changes observed in this study and vocal changes observed in our previous study for the same set of characters (Berry & Brown, 2019).

### Character classification

**Figure 1.**
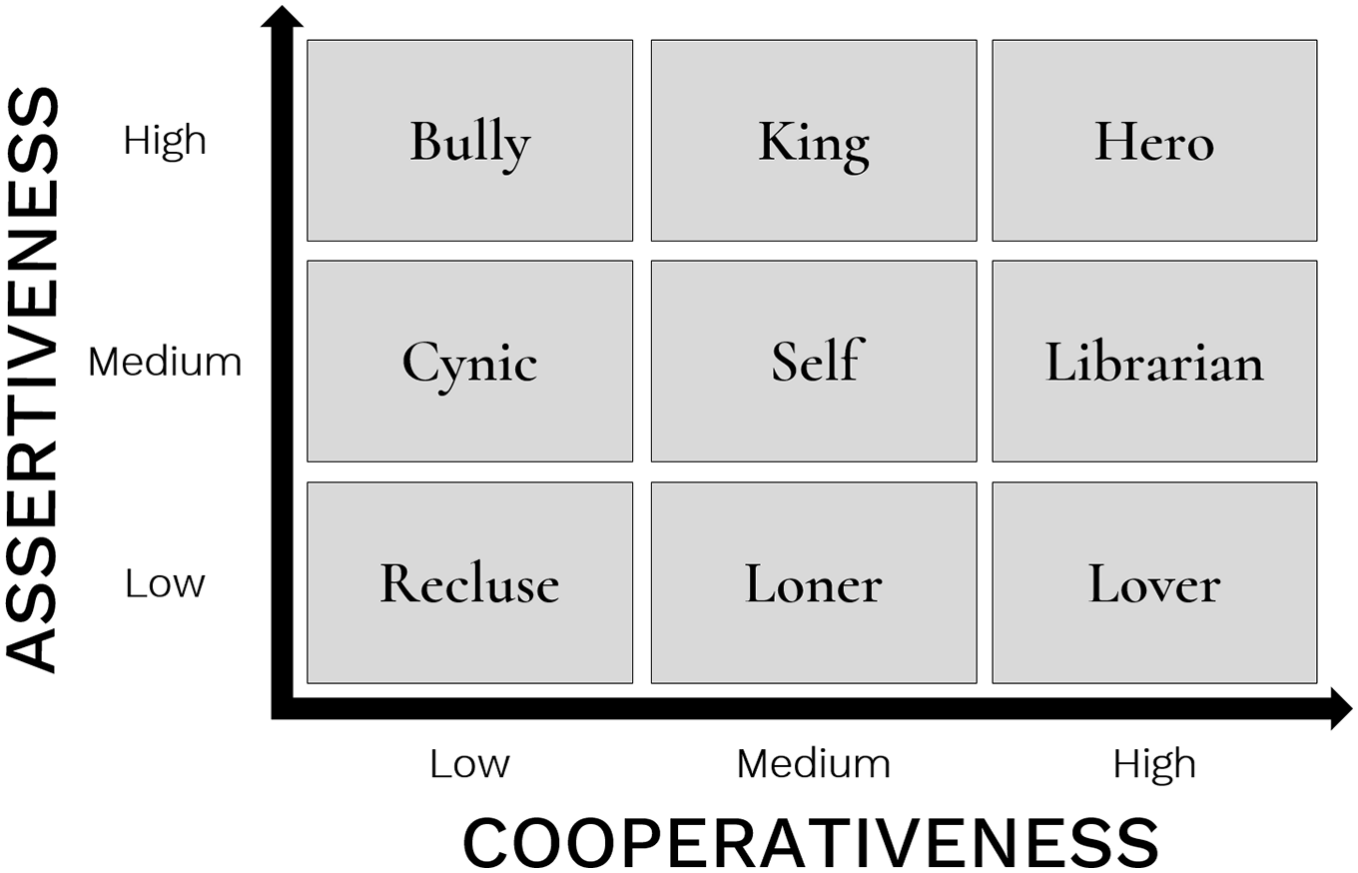
Character scheme. The figure shows the nine stock characters used in the study, as organised into a 3 × 3 scheme based on a crossing of three levels of assertiveness and cooperativeness, respectively. The scheme is adapted from Berry and Brown (2019).

A first step towards quantifying the facial correlates of character during acting is to develop a means of classifying characters dimensionally. In our earlier study (Berry & Brown, 2017), we presented a proposal for a systematic classification of literary characters based on personality dimensions, using a modification of the Thomas–Kilmann Conflict Mode Instrument used in applied studies of personality (Kilmann & Thomas, 1975, 1977; Thomas, 1992). The Thomas–Kilmann scheme classifies personality along the two statistically independent dimensions of assertiveness and cooperativeness. Assertiveness can be understood as the tendency to satisfy one’s personal concerns, whereas cooperativeness can be understood as the tendency to satisfy another’s concerns (Kilmann & Thomas, 1975, 1977; Thomas, 1992). We conducted a character-rating study in which participants rated 40 stock characters with respect to their assertiveness and cooperativeness, and the results demonstrated that these ratings were statistically independent (i.e., orthogonal). The scheme is shown in Figure 1, in which a crossing of three levels of assertiveness and three levels of cooperativeness results in nine character types. In our previous study (Berry & Brown, 2019), we employed this scheme to examine the vocal correlates of character portrayal by analysing the vocal prosody of professional actors as they performed portrayals of the nine characters shown in the figure. The results revealed strong effects of character assertiveness on vocal prosody—both pitch and loudness increased monotonically with increasing assertiveness—but relatively weak effects of character cooperativeness on the voice.

### Objectives

The primary objective of this study was to follow up on our prosody findings by looking at the facial correlates of character portrayal in an experimental study for the first time. We did this using professional actors and a high-resolution 3D motion capture set-up in a black-box performance laboratory. A group of 24 actors performed a semantically neutral script (see Supplemental Appendix A) while portraying eight stock characters and the self, as per the assertiveness/cooperativeness scheme shown in Figure 1. They also performed the script while depicting nine basic emotions so as to allow us to examine the relationship between character and emotion. From a group of 20 facial markers employed in the study, we created 4 facial segments from a subset of 7 selected markers to explore the mean expansion and/or contraction of the brow, eyebrow, lips, and jaw, respectively, across the roughly 2-min performance trials. The four segments that were selected for this study provide accessible information about vertical and horizontal movements in both the upper and lower portions of the face.

To control for movement of the face due to speech articulation, we subtracted out the neutral emotion condition from each character or emotion trial, and then normalised this difference relative to the neutral condition. The overall aims of the study were (1) to examine the expansion/contraction of the four facial segments as a function of character assertiveness and cooperativeness, (2) to examine the same segments as a function of emotional valence and arousal, (3) to analyse the relationship between stock characters and basic emotions, and (4) to use the vocal data from our previous study (Berry & Brown, 2019) to look multimodally at the correlation between facial expression and vocal prosody during acting. Given that assertiveness showed a much larger effect than cooperativeness in our previous study, we predicted that we might observe a similar effect for the face as well. In addition, given that both Scherer and Ellgring (2007b) and Thompson and Russo (2007) observed that vocal pitch-register correlated with upward movement of the brow, we predicted that we might see a correlation between vocal pitch and expansion of the brow segment; Thompson and Russo (2007) also found a correlation between vocal register and mouth opening. Finally, we carried out an exploratory analysis to examine whether there was a relationship between stock characters and basic emotions. Specifically, we conducted a multimodal, multivariate analysis in which we predicted that intuitively associated pairings of characters and emotions (e.g., hero and proud, loner and sad, bully and angry) would cluster together, as based on a combination of the actors’ vocal and facial gestures.

## Methods

### Participants

Participants and procedures are similar to those reported in Berry and Brown (2019). Twenty-four actors (14 males; 20–63 years; *M*_Age_ = 42.5 ± 14 years) were recruited for the experiment through contact with theatre companies and academic theatre programmes in the local and surrounding areas. All actors spoke English either as their native language or fluently as their second language (*n* = 1). They had a mean of 27.5 ± 14.3 years of acting experience. Fourteen held degrees in acting, and two were pursuing degrees in acting at the time of the experiment. More than half of the participants (i.e., 17 of the 24) self-identified as professional actors. All participants gave written informed consent and were given monetary compensation for their participation. The study was approved by the McMaster University Research Ethics Board.

### Motion capture

The experiment took place on the stage of a black-box performance laboratory, where the actors performed the tasks facing an empty audience. The experimenter was located in a control room behind the audience section, and the actors could not see him while performing. The 3D motion-capture recordings of the actors’ facial expressions and body gestures were acquired using a Qualisys motion capture system. Sixteen Qualisys Oqus 7 infrared cameras were used to track marker movement in 3D at a sampling rate of 120 Hz. The participants were equipped with 61 passive markers that were placed on key landmarks on their face and body to provide bilateral full-body coverage (20 markers on the face, 37 on the torso and limbs, and 4 on the head via a cap). For the face, marker placement was chosen to correspond to the general locations of key facial AUs (e.g., AUs 1, 2, and 4 for the upper face, and AUs 11, 12, 23, 26, and 27 for the lower face; see the studies by Barrett et al. (2019) and Rosenberg & Ekman (2020)).

### Characters and emotions

Nine stock characters were performed by the actors, as established by the 3 × 3 (assertiveness × cooperativeness) classification scheme that was validated in the study by Berry and Brown (2017) and was implemented in the vocal analysis by Berry and Brown (2019) (see Figure 1). In addition to the characters, we selected eight basic emotions for the actors to perform, as based on previous studies with actors (see Introduction). The selected emotions were happy, sad, angry, surprised, proud, calm, fearful, and disgusted, with neutral serving as a baseline condition. We sought to create an approximate dimensional analysis of these emotions (Russell, 1980), rather than examine them individually. We, therefore, grouped these basic emotions into a 2 × 2 scheme according to their valence and arousal as follows: positive valence + high arousal (happy, proud, surprised), negative valence + high arousal (angry, fearful, disgusted), positive valence + low arousal (calm), and negative valence + low arousal (sad; see Supplementary Figure 1). The order of presentation of the 9 characters and 9 emotions was randomised across the 18 trials for each participant.

### Performance script

A semantically neutral monologue script was created for the study that the actors memorised in advance of the experiment (see Supplemental Appendix A). It was comprised of 7 neutral sentences (*M* = 6 ± 1.4 words/sentence) derived from a set of 10 validated linguistically neutral sentences (Ben-David, van Lieshout, & Leszcz, 2011; Berry & Brown, 2019). The script was structured such that a small narrative was presented (i.e., someone walking into a room and listing the items that they see), but with no indication of emotion or interpersonal interaction. The same monologue was used for all 18 character and emotion trials. Each trial lasted approximately 2 min, and the full set of trials lasted no more than 45 min. At the end of the session, the actor was debriefed and compensated. To acquire a measurement of the participant’s normal conversational expressions separate from the acting trials, the experimenter (M.B.) indicated that a piece of equipment required additional calibration, and requested that the participant recite the neutral script in a conversational manner as part of the calibration procedure. This recording was used as the control-self to compare against the performed-self from the acting trial (see the “Results” section).

### Data processing and cleaning

Passive reflective-marker movements for the face were recorded in 2D and reconstructed into 3D for analysis. For quality 2D-to-3D data reconstruction, each marker had to be captured by a minimum of two cameras at any given time point. The 16 cameras that were used were placed optimally above and across the designated performance space to track the movement of each marker within the measurement volume. The 2D-tracked motion data were processed using the Qualisys reconstruction algorithm. This proprietary algorithm takes 2D points from each ray of capture from each camera and sorts the data into 3D points and movement trajectories, which can then be used to create an analysable 3D model with the user interface (UI; Qualisys AB, 2006). Following this, each trial was cleaned manually using the 3D model via the UI. For cleaning, each marker and movement trajectory began as an unidentified value, which was then identified manually and provided a label over the course of the trial. Any extraneous trajectories (e.g., noise, errors, reflective artefacts, unassigned or outlying markers) were excluded. No interpolation was done. If there were gaps in the 3D motion trajectory reconstruction, they were not filled. Instead, the data from that particular marker was temporally omitted. This was done to prevent the system from incorrectly interpolating and/or skewing the motion data and thereby artificially changing the mean. The cleaned X coordinates (anterior–posterior movement), Y coordinates (right–left movement), and Z coordinates (superior–inferior movement) were exported into data tables for further analysis.

### Transformation of variable parameters

**Figure 2.**
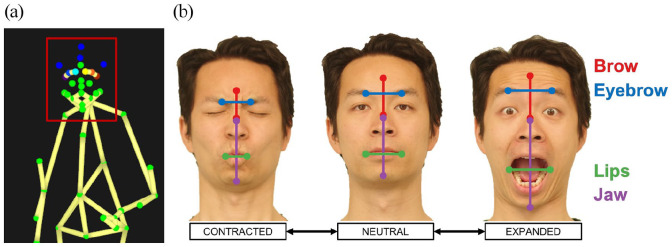
Facial segments. (a) A digital representation of the marker placement on the face as outlined by the red square. Four facial segments were created between marker pairs by calculating the Euclidean distance between them. (b) A visual representation of the four facial segments analysed in the study (brow, eyebrow, lips, and jaw), as well as the approximate locations of the facial markers used to measure them. Facial contraction (left) and expansion (right) are shown with reference to a neutral resting state (centre). Photos are courtesy of author MB. The model gave consent for the use of these photographs.

The variables of interest in this study are those related to expansion and contraction of facial segments. From the 20 available facial markers, we selected a subset of 7 for the current analysis: the brow (corresponding approximately to AUs 1 and 2), the left and right eyebrow (AU 4), the bridge of the nose, the left and right lip corners (AUs 11, 12, and 23), and the jaw (AUs 26 and 27; see Barrett et al., 2019; Rosenberg & Ekman, 2020). Pairs of markers were combined into four facial segments whose expansion and contraction were measured in three-dimensional space, as shown in Figure 2. These segments permitted an analysis of (1) vertical raising and lowering of the brow, (2) horizontal movement of the eyebrows towards or away from the midline of the nose, (3) horizontal movement of the corners of the lips to or away from the midline of the mouth, and (4) vertical lowering and raising of the jaw. Each segment’s length was calculated from the raw exported *x*, *y*, and *z* coordinates for the pair of contributing markers using the following formula for Euclidean distance:

$$d\;=\;\surd {\left(x_{2}-\;x_{1}\right)}^{2}+\;{\left(y_{2}-\;y_{1}\right)}^{2}+\;{\left(z_{2}-\;z_{1}\right)}^{2}$$

where *d* is the Euclidean distance (i.e., the absolute geometric distance) between two points in 3D space, and *x*, *y*, and *z* are the 3D coordinates of a single sample at time (2) and time (1), respectively. A time series of the Euclidean distance for each facial segment was then created for each approximately 2-min trial. The mean segment length across this time series was calculated for the four facial segments using the following formula:

$$M_{d_{ij}}=\sum d_{ij}/\mathrm{sr}*\left(t_{ij}\right)$$

where *M_d_* is the mean Euclidean distance in millimetres between marker pairs over the length of the entire trial (i.e., the mean segment length), *d* is the segment length, sr is the motion capture sample rate (i.e., 120 Hz), and *t* is the time in seconds of the entire trial. This resulted in a total of four parameters for the analysis (i.e., four facial-segment means). Each facial-segment parameter mean was extracted for each participant (*i*) for each character or emotion condition (*j*).

### Correcting for speech-related movements and facial-size differences

A “percent change” transformation was applied to the four segmental parameter means in order to eliminate the impact of speech articulation on facial movements—especially for the jaw and lip segments—and to remove any bias caused by subject-related differences in facial size. This was carried out by subtracting the mean segmental lengths for the neutral emotion condition (i.e., speaking the script devoid of any emotion or character) from the means for each character and emotion trial, as per the following formula:

$$\%\;\mathrm{change}=100\times \left(\left(M_{d[\mathrm{performance}]}-\;M_{d[\mathrm{neutral}]}\right)/M_{d[\mathrm{neutral}]}\right)$$

where the percentage change is the difference between the mean Euclidean distance for a participant’s given performance condition (character or emotion) and the participant’s neutral emotion condition, scaled to that neutral condition, and then multiplied by 100. As a result, all data for the characters and emotions are reported as a percentage change relative to the neutral emotion condition. Following this correction, each parameter was visually screened for extreme outliers, of which none were found.

### Univariate analyses

Each of the four transformed parameters was analysed using a linear mixed-effects regression model (LMER) with maximum likelihood estimation using the *lme4* package in *R* (Bates, Mächler, Bolker, & Walker, 2015; R Core Team, 2013). For the character trials, a two-way repeated measures analysis of variance (ANOVA) was conducted for each of the four parameters. For the character trials, the two orthogonal dimensions of assertiveness and cooperativeness were treated as within-subject factors, while subject was treated as the random effect. For the emotion trials, the two approximated dimensions of valence and arousal from the circumplex model of emotion were treated as within-subject factors (i.e., fixed effects), while subject was treated as the random effect. The neutral emotion condition—which was used as the baseline condition for data normalisation—was not included in either of these analyses, but was only included in the multivariate analysis, described below. The final sample for the univariate analysis was therefore *n* = 216 for characters (9 characters × 24 participants) and *n* = 192 for emotions (8 emotions × 24 participants). For the reporting of *F* values, we used type III sums of squares with Satterthwaite approximations for degrees of freedom. Statistical significance levels were set to α < .05, and adjustments for repeated testing of the group of four segmental parameters were made using Bonferroni corrections (i.e., α/4 for each segment, resulting in a new threshold of α < .0125; Berry & Brown, 2019; Goudbeek & Scherer, 2010). The significance of statistical analyses and the estimation of effect sizes examine how much of the model’s variance is explained by the fixed effects only (*R*_marg_^2^) and how much of it is explained by the complete (fixed + random effects) model (*R*_cond_^2^). These were calculated using the *afex* package in R (Singmann et al., 2016).

### Correlation analysis

Correlations between the segmental parameters and the vocal parameters of pitch (in cents) and loudness (in decibels) were carried out using the vocal data reported in Berry and Brown (2019) using the same trials. Statistical significance was set to α < .05, and adjustments for repeated testing of the group of four segmental parameters were made using Bonferroni corrections (i.e., α/4 for each segment, resulting in a new threshold of α < .0125; Berry and Brown, 2019; Goudbeek & Scherer, 2010).

### Multivariate analysis

To look at the relationship between characters and emotions, we carried out a principal components analysis (PCA) using the principal function in the *psych* package in *R* (R Core Team, 2013; Revelle, 2017) in which data from the characters and emotions were combined into a single analysis. This included the 4 facial parameters described above and the 12 vocal parameters described in the study by Berry and Brown (2019). Due to the differing scales employed for each parameter, all parameter scores were normalised within-subject using *z*-scores prior to analysis. Normalising the data also corrected for the presence of extreme outliers. Two librarian trials were omitted due to whispering, and so these missing data were imputed using “similar case imputation” of the mean. The final sample for the multivariate analysis was *n* = 432 (9 characters + 9 emotions × 24 participants). Cartell’s Scree Test and the Kaiser Criterion suggested that the first two principal components were sufficient for extraction and interpretation (Berry & Brown, 2019; Cangelosi & Goriely, 2007; Dunteman, 1989). The first two components were, therefore, rotated using the *varimax* rotation in the *psych* package and extracted for interpretation.

## Results

### Univariate analyses

**Figure 3.**
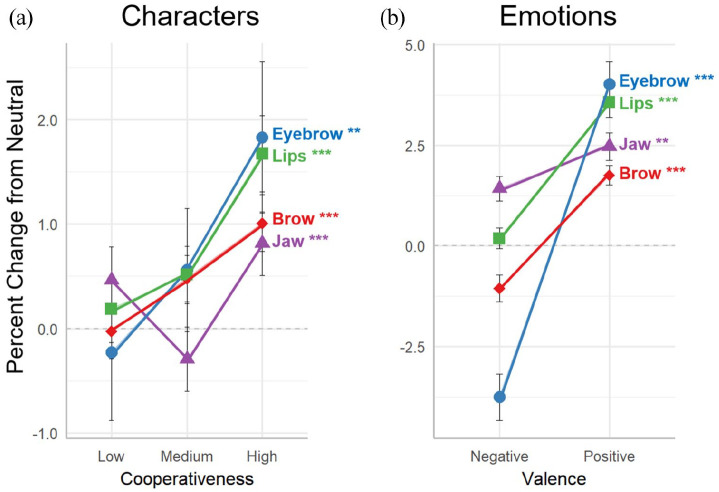
Facial correlates of cooperativeness and valence. Effect of (a) character cooperativeness and (b) emotional valence on the parameter means of the four facial segments. Values for each facial segment are the percentage change relative to the participant’s neutral emotion condition, which corrects for speech articulation and the diversity of facial dimensions across participants. Error bars indicate the standard error of the mean. Significance values are from a linear mixed-effects regression model for the main effects of the character and emotion dimensions. **p < .01, ***p < .001. See Table 1 (character) and Supplementary Table 1 (emotion) for full descriptions.

**Figure 4.**
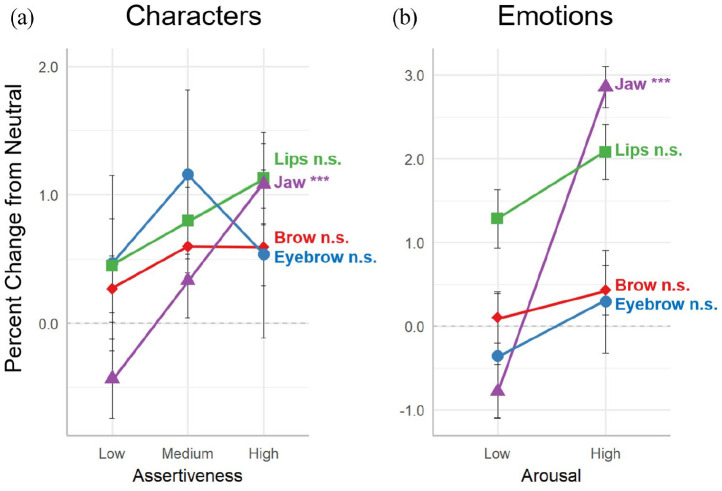
Facial correlates of assertiveness and arousal. Effect of (a) character assertiveness and (b) emotional arousal on the parameter means of the four facial segments. Values for each facial segment are the percentage change relative to the participant’s neutral emotion condition, which corrects for speech articulation and the diversity of facial dimensions across participants. Error bars indicate the standard error of the mean. Significance values are from a linear mixed-effects regression model for the main effects of the character and emotion dimensions. ***p < .001, n.s., not significant. See Table 1 (character) and Supplementary Table 1 (emotion) for full descriptions.

Table 1 provides a summary of the analyses of variance conducted on the LMER model for the four facial segment means across the nine characters. The characters are collapsed across the three levels (low, medium, and high) of each personality dimension (cooperativeness and assertiveness), and the main effects of each dimension, as well as their interaction, are presented in the table. The results for the mean displacement of the four facial segments are shown in Figure 3 (for character cooperativeness and emotional valence) and Figure 4 (for character assertiveness and emotional arousal). The left panel of each figure provides results for the character dimension, and the right panel for the related emotion dimension. All results are controlled for the speech-related movements of the neutral emotion condition.

**Table 1.** ANOVA results for the character dimensions.

Segment	Direction	Effect type	SumSq	MeanSq	NumDF	DenDF	*F*-value	*p*-value	Sig.	*R*_marg_^2^	*R*_cond_^2^
Brow	Vertical	Coop	38.40	19.20	2	192	7.83	.001	***	.08	.48
		Assert	5.19	2.59	2	192	1.06	.349	NS		
		Coop × Assert	34.41	8.60	4	192	3.51	.009	**		
Eyebrows	Horizontal	Coop	155.49	77.75	2	192	4.82	.009	**	.04	.61
		Assert	20.83	10.41	2	192	0.65	.526	NS		
		Coop × Assert	192.41	48.10	4	192	2.98	.020	*		
Lips	Horizontal	Coop	87.88	43.94	2	192	10.41	.000	***	.26	.41
		Assert	16.83	8.41	2	192	1.99	.139	NS		
		Coop × Assert	295.57	73.89	4	192	17.50	.000	***		
Jaw	Vertical	Coop	46.13	23.06	2	192	8.45	.000	***	.17	.34
		Assert	82.27	41.13	2	192	15.06	.000	***		
		Coop × Assert	21.39	5.35	4	192	1.96	.102	NS		

ANOVA: analysis of variance; SumSq.: sum of squares; MeanSq.: mean squares; NumDF: numerator degrees of freedom; DenDF: denominator degrees of freedom; Sig.: significance level; Coop: cooperativeness; Assert: assertiveness; NS: not significant, *R*_marg_^2^: marginal *R* squared; *R*_cond_^2^: conditional *R* squared.

Note: Summary of the repeated measures analysis of variance (ANOVA) for each segment, after controlling for speech-related movements using the neutral emotion condition. A linear mixed-effects regression analysis (LMER) was computed, with subjects listed as the random effect and the two character dimensions (cooperativeness and assertiveness) as the fixed effects. The ANOVA table includes type III sum of squares using Satterthwaite approximation for degrees of freedom. Measures of effect size indicate how much of the model’s variance is explained by the fixed effects only (*R*_marg_^2^), and how much of it is explained by the complete (fixed + random effects) model (*R*_cond_^2^).

* *p* < .05, **p < .01, ***p < .001.

#### Cooperativeness

There were significant main effects of cooperativeness on both the vertical and horizontal segments of the upper and lower face. These results reflect an increase in mean segment expansiveness with increasing character cooperativeness. Monotonic increases in segment expansion were most pronounced for the brow and lips (Figure 3a), with a smaller but significant effect for the eyebrow. The effect of character cooperativeness on jaw expansion was significant but non-monotonic, showing a “V” pattern of greater expansion for low and high cooperativeness than for medium cooperativeness. Next, when the basic emotions were organised in a manner that is most analogous to character cooperativeness—namely according to emotional valence—parallel results were obtained, with significantly greater expansion in all four facial segments for positive-valence emotions than negative-valence emotions (Figure 3b). Supplementary Table 1 provides the ANOVA and LMER analyses for the emotion data.

#### Assertiveness

The segments that showed the greatest mean expansiveness for character cooperativeness showed null effects for character assertiveness (Figure 4a). This applied to the brow, eyebrow, and lips. By contrast, the jaw showed monotonic increases in expansiveness with increasing assertiveness (Figure 4a). When the basic emotions were organised in a manner that is most analogous to character assertiveness—namely according to emotional arousal—parallel results were obtained, with null effects for the brow, eyebrow, and lips, but a significant expansive effect for the jaw segment (Figure 4b). Overall, a dissociation between cooperativeness and assertiveness was observed for the four segments, with the brow, eyebrow, and lips showing significant and monotonic effects for cooperativeness (and emotional valence) and the jaw for assertiveness (and emotional arousal).

#### Additional vertical segments

Beyond the hypothesised effects, we ran additional exploratory analyses on two upper-face and two lower-face segments. We analysed two vertical segments for the eyebrow, going from the bridge of the nose to the left or right inner eyebrow, respectively. Similarly, we analysed two vertical segments for the lips, going from the bridge of the nose to the left or right lip corner, respectively. The results are shown in Supplementary Table 2. The vertical eyebrow segments mirrored the horizontal segment by showing monotonic increases across the levels of both cooperativeness and valence, as well as null effects for both assertiveness and arousal. The vertical lip segments were more complex, showing minimal effects for both cooperativeness and valence, despite the strong effect of the horizontal segment. In addition, while these segments showed a similar effect to the horizontal segment for arousal, they showed a contrasting profile to the null effect of the horizontal lip segment on assertiveness, thereby mirroring the monotonic increase seen with the jaw segment.

### Correlations with vocal parameters

Table 2 presents correlations between the mean displacements of the four facial segments and both vocal pitch (in cents) and loudness (in decibels) for the same trials (see Supplementary Figures 2 and 3 for the character-condition regressions and Supplementary Figures 4 and 5 for the emotion-condition regressions). As per one of our predictions, based on the findings of Scherer and Ellgring (2007b) and Thompson and Russo (2007), a significant correlation was found between vocal pitch and upward movement of the brow, although this effect was only significant for the emotions (but not the characters) after correcting for multiple comparisons (α = .0125; Berry & Brown, 2019). The most significant finding of the analysis was a correlation between jaw expansiveness and both pitch and loudness in the voice (see also Thompson & Russo, 2007, for singing), an effect that was seen for both the characters and the emotions, although more strongly for the emotions. The results remained significant after correcting for multiple comparisons. These findings point to a synergy between the voice and the face during acting, one that applies to both character portrayal and the expression of emotions. It is important to keep in mind that the facial data were transformed to eliminate speech-related movements separate from character and emotion. Hence, the observed jaw/voice and brow/voice correlations are present above and beyond an influence of speech articulation alone and are thus performance-related effects.

**Table 2.** Correlations between facial and vocal parameters.

Segment	Regressor	Condition	*R*	*R* ^2^	*p*-value	Sig.	Low conf.	High conf.	DF	*T*-value
Brow	Pitch	Character	.14	.02	.040	*	0.01	0.27	212	2.1
		Emotion	.28	.08	.000	***	0.15	0.40	214	4.3
	Loudness	Character	.14	.02	.041	*	0.01	0.27	212	2.1
		Emotion	.25	.06	.000	***	0.12	0.37	214	3.7
Eyebrows	Pitch	Character	−.04	.00	.548	NS	−0.17	0.09	212	−0.6
		Emotion	.15	.02	.024	*	0.02	0.28	214	2.3
	Loudness	Character	.08	.01	.246	NS	−0.06	0.21	212	1.2
		Emotion	.17	.03	.011	*	0.04	0.30	214	2.6
Lips	Pitch	Character	.18	.03	.008	**	0.05	0.31	212	2.7
		Emotion	.28	.08	.000	***	0.15	0.39	214	4.2
	Loudness	Character	.16	.03	.020	*	0.03	0.29	212	2.3
		Emotion	.17	.03	.014	*	0.04	0.29	214	2.5
Jaw	Pitch	Character	.40	.16	.000	***	0.28	0.50	212	6.3
		Emotion	.74	.55	.000	***	0.68	0.80	214	16.2
	Loudness	Character	.29	.08	.000	***	0.16	0.41	212	4.4
		Emotion	.62	.39	.000	***	0.53	0.70	214	11.6

Sig.: significance level; DF: degrees of freedom; Low conf.: low end of confidence interval; High conf.: high end of confidence interval; NS: not significant.

Note: Pearson’s product–moment correlation, two-tailed, *p < .05, **p < .01, ***p < .001.

### Performed- versus control-self

Our previous study demonstrated that when the self was performed as a character, it showed increases in pitch and loudness compared to the control-self that was done during the instrument calibration (Berry & Brown, 2019). We wanted to examine whether parallel effects would be observed for the face as well. Significant, small-to-moderate expansive effects were seen for the eyebrow, lips, and jaw for the performed-self compared to the control-self (see Supplementary Table 3). These results are consistent with the observation that the actors approached the self in a more performative manner during the acting trials.

### Relationship between characters and emotions

**Figure 5.**
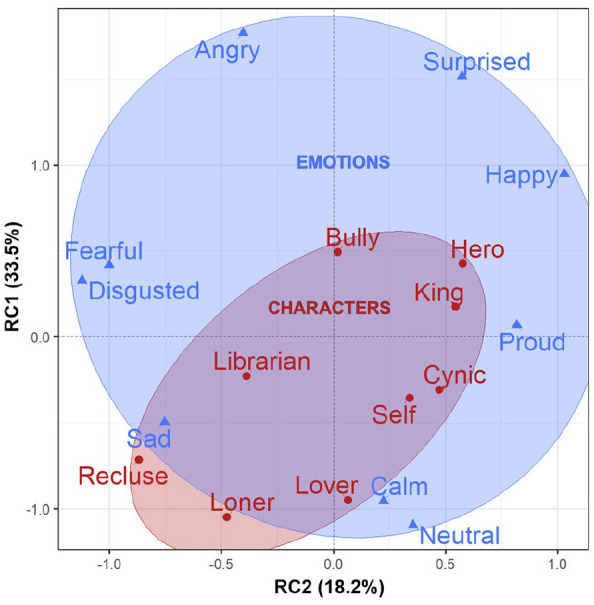
Varimax-rotated principal components plot for the characters and emotions. The characters are colour-coded red, and the red circle contains the space of all characters. The basic emotions are colour-coded blue, and the blue circle contains the space of all emotions. This multimodal analysis combines all of the facial parameters from the current analysis (i.e., the facial segments of the brow, eyebrow, lips, and jaw) with all of the vocal parameters (i.e., pitch, loudness, duration, and timbre parameters) reported in the study by Berry and Brown (2019). Rotated component 1 (RC1) is suggestive of an intensity dimension, while rotated component 2 (RC2) is suggestive of an evaluative or quality dimension. Condition locations are averaged across all participants.

To look at the relationship between characters and emotions, we ran a PCA that combined the modalities of the current facial dataset with our previous vocal dataset (Figure 5). We reduced the parameters of the combined dataset from 16 variables to 2 underlying components that accounted for 52% of the total variance in the combined dataset. These first two principal components were extracted using a *varimax* rotation, with the first rotated component (RC1) accounting for 33.5% of the variance, and the second rotated component (RC2) for 18.2%. We would expect a total of only 12.5% of the variance (i.e., 2/16 variables) if the results were due to chance alone. A summary of the loadings for each rotated component can be found in Supplementary Table 4. These loadings provide information about how to interpret the RCs, as well as the placement of the individual conditions on the plot. Note that each individual condition’s location is the average across all 24 participants.

RC1 is primarily characterised by strong (|0.60| < *x* <|0.79|) to very strong (|0.80| < *x*) positive loadings for jaw expansion, pitch, and loudness. It is additionally characterised by moderately low (|0.35| < *x* <|0.49|) negative loadings for pause duration and shimmer for the voice. These loadings cause the spread of both characters and emotions along RC1, specifically accounting for variation in the levels of character assertiveness and emotional arousal (i.e., intense conditions group on the top, and mild conditions group on the bottom). This suggests that RC1 can be described as representing an *intensity* dimension. RC2 is primarily characterised by moderate (|0.50| < *x* <|0.59|) to strong (|0.60| < *x* <|0.79|) positive loadings for expansion of the brow, eyebrow, and lips. It is additionally characterised by moderately low (|0.35| < *x* <|0.49|) to moderate (|0.50| < *x* <|0.59|) negative loadings for duration and timbre for the voice. These loadings cause the spread of both characters and emotions along RC2, specifically accounting for variation in the levels of character cooperativeness and emotional valence (i.e., negative conditions group on the left and positive conditions group on the right). This suggests that RC2 can be described as representing an *evaluative* or *quality* dimension.

The PCA reveals four important features of the relationship between characters and emotions. First, the emotions themselves approximate the structure of a circumplex model of emotions in the rotated principal component (RPC) dimensional space. Recall that the circumplex is a circular organisation of emotion categories along the two dimensions of valence and arousal, and constitutes the structural analogue of the “core affect” theory of emotion (Posner et al., 2005; Russell, 1980, 1994, 2005, 2009). Hence, the use of a segmental approach to facial expression, combined multimodally with a series of prosodic vocal parameters, leads to a result that resembles the circumplex organisation for emotion posited by Russell (1980). Second, the characters themselves approximate the structure of the 3 × 3 predictive character scheme in the RPC dimensional space. Third, the characters as a whole are contained within the overall emotion space, suggesting that the character portrayals were less extreme from an expressive standpoint than the emotion trials (rather than being distinctively different, which would have placed them in separate clusters from the emotions). Fourth, and perhaps most importantly, the characters are situated in the RPC space in a location that is proximate to or approximating towards the basic emotions that are intuitively associated with such characters. For example, hero and king are proximate to proud, lover is proximate to calm and neutral, recluse and loner are proximate to sad, and bully is approximating towards angry. Overall, the characters are contained within the general space of the emotions and are oriented in such a way as to approximate certain basic emotions intuitively associated with them.

## Discussion

We carried out the first production-based experimental study of the facial correlates of character portrayal, looking at mean trends and variability across a cohort of 24 professional actors. The characters were organised along the two orthogonal personality dimensions of assertiveness and cooperativeness. We found a significant main effect of cooperativeness on facial expression—with monotonic increases in facial expansiveness as the characters were increasingly cooperative in nature—but fewer facial correlates of character assertiveness, the jaw being the main effector to convey this. This is in contrast to our predictions based on the profile that we observed in our previous study of the vocal correlates of character portrayal (Berry & Brown, 2019), in which the strongest prosodic effects were found for assertiveness, rather than cooperativeness. A comparison between the results for the face and voice showed that the jaw was the clearest interface between facial expression and vocal prosody during spoken character portrayals, where increased mouth opening via jaw lowering correlated with increases in both pitch and loudness at the vocal level. Finally, the PCA analysis demonstrated that the stock characters in our two-dimensional scheme were proximate to the basic emotions that one might intuitively associate with these characters, but that the characters overall occupied a subset of the expressive space of the emotions, arguing that, while characters might have associations with particular emotions, they are not limited to these emotions and are not as extreme, from an expressive or performative standpoint, as individual emotions in isolation (Berry & Brown, 2019).

### Analysing facial expression using segments

Most quantitative studies of facial expression examine individual AUs within the face, as seen most notably in methods based on the FACS (Barrett et al., 2019; Bartlett et al., 2005; Ekman & Friesen, 1978a, 1978b; Ekman & Rosenberg, 1997, 2005; Rosenberg & Ekman, 2020). We followed the lead of a number of previous studies in looking at facial segments (Fasel & Luettin, 2003; Hammal et al., 2007; Hammal & Massot, 2010; Kanade et al., 2000; Livingstone et al., 2015; Sandbach et al., 2012; Schmidt et al., 2003; Soyel & Demirel, 2007; Valstar et al., 2007; Yacoob & Davis, 1996; Zhang et al., 2015), instead of the combinatorial activation of facial AUs. In addition, we used a high-resolution motion capture system to examine the expansion and contraction of these segments in 3D, rather than in 2D (for 2D, see the study by Schmidt et al., 2003; for 3D, see the studies by Livingstone et al., 2015; Soyel & Demirel, 2007; and Zhang et al., 2015; for a review, see the study by Zhi et al., 2020). In doing so, we were able to observe monotonic changes in segment expansiveness as a function of dimensional features of the characters and emotions that were analysed in this study. The use of 3D facial segments might serve as a useful complement to studies of AUs that focus on the role of specific muscles in producing facial expressions.

Compared to the level of an AU, a segment deals with a more integrative and more communicative level of facial expression, one that is intuitively understandable for most people. We analysed four segments in this study, two vertical segments (the brow and the jaw) and two horizontal segments (the eyebrows and lips). While the contrast between a happy and sad facial expression can certainly be analysed in terms of the underlying changes in AUs (Barrett et al., 2019; Scherer & Ellgring, 2007a, 2007b), they can also be analysed as changes in the expansiveness of facial segments (Livingstone et al., 2015; Schmidt et al., 2003; Soyel & Demirel, 2007). In particular, happiness is associated with relative expansion in all four of the segments we used, while sadness is associated with relative contraction. The study of segments highlights the fact that, due to the differing innervation patterns of different facial zones by the descending motor system (Wilson-Pauwels et al., 2002), the lower face is activatable in a more lateralised manner than the upper face, allowing people to raise just one cheek or abduct the jaw to one side. It will be important to ground the study of facial expression into particular facial zones (Guha, Yang, Grossman, & Narayanan, 2018), based in part on the innervation pattern of the face by the orofacial motor cortex. This is well-established in the distinction between the Duchenne smile and the fake smile, where the fake smile replicates features of the genuine smile in the lower face, but not the upper face (Carroll & Russell, 1997; Ekman, Davidson, & Friesen, 1990; Ekman, Friesen, & Hager, 2002a, 2002b; Gunnery & Hall, 2014).

### Analysing facial expression in the context of speech

Facial expression was studied here in the context of spoken performance, rather than by looking at the static and posed depictions of emotional expressions that have been prevalent in much of the literature (Barrett et al., 2019; Carroll & Russell, 1997; Ekman & Friesen, 1975, 1976, 1978a, 1978b; Ekman et al., 2002a). This was done by normalising the acting performances to the neutral emotion condition (Zhang et al., 2015), thereby correcting for general articulatory movements of the facial segments while still preserving the facial actions used for character portrayal. This not only allowed for a multimodal analysis of emotional expression between the face and voice, but also a more naturalistic manner of analysing facial expression, since much facial expression occurs in the communicative context of conversation. Indeed, Girard, Cohn, Jeni, Sayette, & De la Torre (2015), in their investigation of spontaneous expressions in non-scripted social interactions, noted that, while automatic AU detection of multiple conversing individuals is possible, the influence of speech on AU classification and intensity is difficult to assess and could not be evaluated using their methodology. Alternative methods of assessing the recruitment of AUs during facial expression and co-articulation have been used, such as assessing their presence or absence via coders while not directly correcting for speech confounds (Benitez-Quiroz, Wilbur, & Martinez, 2016) or analysing facial expression functionally in conjunction with speech (Livingstone et al., 2015). Livingstone and colleagues (2015) found that co-articulated facial movements for happy and sad expressions were relatively similar to their non-speech forms (e.g., happiness exhibited increased movements of the eyebrows, lip corners, and jaw). This highlights the point that facial expression is not purely a spontaneous, static activity, but that it contains important dynamic and communicative information. People engaged in a conversation show a great deal of facial mirroring (Hatfield, Bensman, Thornton, & Rapson, 2014; Hess & Blairy, 2001; Seibt, Mühlberger, Likowski, & Weyers, 2015), a process that is thought to increase the social cohesion of the interlocutors.

The multimodal analysis revealed a series of correlations between the four facial segments and the two prosodic parameters of pitch and loudness analysed previously (Berry & Brown, 2019). The results showed that, even after controlling for speech production in the neutral control condition, there were significant correlations between all facial segments and the vocal parameters. The most significant ones were between jaw lowering and increases in both pitch and loudness. These results corroborated previous research on the correlation between mouth opening and vocal pitch (Thompson & Russo, 2007), but extended it to include vocal loudness as well. In addition, we observed that increases in pitch were correlated with increases in brow raising, in support of previous findings (Scherer & Ellgring, 2007b; Thompson & Russo, 2007). These multimodal correlations were observed in both the character and emotion conditions, although the correlations for emotions were larger, in keeping with the observation from the PCA analysis that the performances of the emotions were more extreme than were those of the characters. Taken together, these results reveal the prospects of analysing facial expression in the naturalistic context of speaking, as well as the benefits of carrying out multimodal analyses between facial expression and vocal prosody.

### A dimensional approach to characters and emotions

Our previous study of vocal prosody employed the 2D scheme for classifying literary characters developed by Berry and Brown (2017) to examine the prosodic correlates of character portrayal in the same cohort of actors used here. The results demonstrated significant effects of character assertiveness on all prosodic parameters, but few effects of character cooperativeness on such parameters. The current analysis of facial expression showed very nearly the opposite pattern, with significant monotonic effects of cooperativeness on the expansiveness of three of the four facial segments, but an effect of assertiveness on the one segment that did not show a monotonic trend for cooperativeness, namely the jaw segment. These results reveal an important complementarity between the voice (assertiveness) and face (cooperativeness) in the communication of expressive information, as well as one between the facial effectors that linearly convey cooperativeness (the brow, eyebrow, and lips) and the one that linearly conveys assertiveness (the jaw). As mentioned above, the jaw showed an important cross-modal relationship with the voice via correlations with vocal pitch and loudness. These results are consistent with the idea—based in part on the contrastive appearance between the Duchenne smile and the fake smile—that the upper part of the face is a more honest indicator of felt emotions, whereas the lower face can be more effectively recruited to generate fake expressions (Carroll & Russell, 1997; Ekman et al., 1990, 2002a, 2002b; Gunnery & Hall, 2014).

These results provide further support for our 2D classification of literary characters (Berry & Brown, 2017), as well as for the relevance of assertiveness and cooperativeness as salient personality dimensions by which characters can be meaningfully classified, as based on the Thomas–Kilmann Conflict Mode Instrument (Kilmann & Thomas, 1975, 1977; Thomas, 1992). We extended this notion of dimensionality by organising the basic emotions included in the study in a dimensional manner to examine the character/emotion relationship. When we organised the emotions according to the dimensional scheme of the circumplex, we were able to recreate the circumplex structure in the PCA analysis using dynamic segmental data for the face. In addition, we observed striking parallels between the character dimension of cooperativeness and the emotion dimension of valence in the univariate analyses, as well as a parallel between the character dimension of assertiveness and the emotion dimension of arousal, a relationship that was alluded to but not analysed in a direct manner in our previous study (Berry & Brown, 2019). These results suggest that cooperativeness and valence collectively comprise a quality factor, whereas assertiveness and arousal collectively comprise an intensity factor. They also suggest that the face might be the preferred modality for conveying expressive quality, while the voice might be the preferred modality for conveying expressive intensity, implying a complementarity between these two effector systems. These results point to the advantage of looking at emotions and characters in a dimensional manner, as well as their application for analysing the character/emotion relationship.

The PCA analysis revealed that characters were often located in PC space proximate to particular emotions that would be intuitively associated with them, such as the king with proud, and the recluse with sad. Such results suggest that actors use facial and vocal parameters in a multimodal manner to contrastively depict both characters and emotions. However, the characters as a whole occupied only a subset of the expressive space of the emotions, suggesting that the portrayals of emotion were more extreme from an expressive standpoint than were the portrayals of characters. Another way of thinking about this is that, while characters may indeed have associations with specific emotions, they are not equivalent to these emotions. They have more complexity to them, leading to greater nuance in performance by actors.

### Personality dimensions and social inferences from faces

In line with this work, previous research on personality-trait and social evaluation of faces has found similar applicability in a dimensional approach. More specifically, researchers have demonstrated that the dimensions of dominance and trustworthiness are robust at approximating trait judgements when evaluating static faces during first impressions (Oosterhof & Todorov, 2008, 2009; Sutherland et al., 2013; for a review see the study by Todorov, 2008). Aspects of a person’s character or personality have been shown to be inferable from non-dynamic structural facial cues. For example, the dimension of trustworthiness is correlated with changes in expression, specifically valence signalling in approach/avoidance behaviours, while the dimension of dominance is correlated with changes in identity, specifically changes in perceived gender and maturity, signalling physical strength/weakness/capability (Oosterhof & Todorov, 2008; Sutherland et al., 2013; Todorov, 2008; Vernon, Sutherland, Young, & Hartley, 2014). Our dimension of assertiveness intuitively aligns with dominance. Indeed, interpretations of assertiveness and confidence are linked to perceived dominance in faces, validating this connection (Hassin & Trope, 2000; Vernon et al., 2014). Our dimension of cooperativeness intuitively aligns with trustworthiness. Variations in trustworthiness seem to be signalled through changes in non-dynamic facial compositions that mimic the expression of emotions, with more trustworthy faces resembling positive emotions like happiness (Oosterhof & Todorov, 2008, 2009; Sutherland et al., 2013; Todorov, 2008; Vernon et al., 2014). Our work broadens this research area by utilising comparable personality dimensions while focusing on a more dynamic and multimodal methodological approach.

### Applications

This work has important applications to a number of areas. These include clinical areas (e.g., behaviour therapy, drama therapy, and simulated care/training), commercial uses (e.g., performance art, consumer-based advertising, body language monitoring, multimedia, and robotics), and entertainment (e.g., storytelling, video games, stage and screen performance; Zhi et al., 2020). An additional application is towards the establishment of a scientifically supported approach to acting methods that is grounded in multimodal expression (Berry & Brown, 2019; Kemp, 2012; Konijn, 2000). Indeed, a segmental view of facial expression allows for a quantification of behaviours that actors can meaningfully incorporate into their performances, either implicitly (e.g., improvisation; Halpern, Close, & Johnson, 1994; Spolin & Sills, 1999) or with training (e.g., acting education; Benedetti, 2012; Brestoff, 1995; Mirodan, 2019). In addition, a scientifically grounded approach could provide testable psychological benefits in areas like theory-of-mind, empathy, and emotion regulation (Goldstein, 2009; Goldstein & Bloom, 2011). Finally, there is an important application of this work to the current interest in embodied cognition and how people can modulate their emotions and the presentation of the self in everyday circumstances through targeted changes to their body, including their facial expressions (Glenberg, 2010; Kemp, 2012; Niedenthal, Winkielman, Mondillon, & Vermeulen, 2009; Scott, Harris, & Rothe, 2001; Shapiro, 2019; Winkielman, Niedenthal, Wielgosz, Eelen, & Kavanagh, 2015). The dramaturgical perspective in social psychology argues that social behaviour is akin to a form of theatre and stagecraft (Goffman, 1959; Shulman, 2017), and that the analysis of social behaviour can benefit from a view from acting theory.

### Limitations

While exploratory, this work has a number of important limitations. A limited number of characters and emotions were used as the functional units of analysis in the study. However, these functional units were performed by a large and diverse group of actors of various trainings, ages, and genders. The ecological validity of the work could be increased by having the actors do their performances in front of an audience. Similarly, the actors could be presented with the characters in advance of the experiment, allowing them to produce more rehearsed and polished interpretations. The character/emotion relationship could be examined in greater detail by creating explicit pairings between characters and emotions, for example by comparing a proud king to an angry king to a sad king, a concept that we have referred to as *ethotypes* of a character (Berry & Brown, 2017). Even though stock characters are thought of as prototypes having relatively fixed traits, the use of different emotion pairings for a given character could highlight the character’s state-dependent features as well. This would be especially important in looking beyond stock characters towards complex dramatic characters. For example, Romeo is initially a happy-go-lucky romantic (i.e., a lover) who falls in love with a girl at a party, but later becomes an anguished fugitive (i.e., a recluse) when he avenges the death of his best friend by killing a member of the rival group. An actor will externalise very different facial expressions, vocal prosodies, and body gestures when playing the balcony scene with Juliet than when playing the duel scene in which Romeo kills Tybalt and flees his home city.

Previous work on performance using actors has made use of automatic and computationally driven 2D and 3D feature-extraction methods, allowing for greater data acquisition and analysis than was possible with our methods (Rosenberg & Ekman, 2020; Soyel & Demirel, 2007; Zhang et al., 2015; for surveys on different types of feature extraction, see the studies by Sandbach et al., 2012 and Zhi et al., 2020). The more limited number of markers used in 3D motion capture systems compared to 2D digital mesh overlays is offset by motion capture’s higher fidelity in all three dimensions, increased sampling rate, and increased resolution (Qualisys AB, 2006). Indeed, Livingstone et al. (2015) took advantage of this increased fidelity by carrying out a translation of the 3D reference points to create a six-degree-of-freedom (6 DOF) quantitative facial analysis of singers. Their 6 DOF analysis included facial-marker movement in the three cardinal planes, as well as yaw, pitch, and roll for the head and neck. This is in contrast to the type of segmental analysis that we carried out, which has 1 DOF and which Livingstone et al. (2015) employed to examine expansion of the jaw. We argue that a 1-DOF segmental analysis of facial expression is readily interpretable in a way that can be easily disseminated in applied uses like actor training and behaviour therapy.

### Acting beyond gesture

During dramatic acting, the character’s cognitions, emotions, perceptions, and actions are all present in the actor’s body, purposefully manifested and constantly manipulated through changes in their gestural signals. Looking beyond production per se, recent research has shown that perceptions of the states and traits of others are influenced by observed inner bodily signals (Barrett, 2017; Galvez-Pol, Antoine, Li, & Kilner, 2020). For example, people can detect physiological changes such as heart rate through observation of colour changes in the face and neck of another person (Galvez-Pol et al., 2020). The “theory of constructed emotion” posits that emotions are contextual, holistic, and grounded in embodied phenomena (Barrett, 2017). If emotions are embodied, it follows that physiological signals may be present during an actor’s character portrayal that, when perceived by an audience, provide additional expressive information. Future production-focused research should examine (1) if there are indeed physiological correlates of character portrayal during dramatic acting, (2) if physiological correlates contrastively differentiate between characters in a manner similar to gestural correlates, (3) if physiological correlates mediate/moderate the gestural correlates of acting, and (4) whether actors consciously control or unconsciously signal physiological correlates when portraying different characters. Future perception-focused research should endeavour to determine the impact of the gestural and physiological correlates of acting on an audience’s experience.

## Conclusion

We carried out the first experimental production study of the facial correlates of character portrayal in professional actors. We applied a 3D segment-based approach for measuring facial changes during performance, and observed that such changes were more reliable indicators of the cooperativeness than the assertiveness of a character, a complementary finding to our previous vocal findings, which showed a stronger relationship of prosody with character assertiveness. Significant correlations were observed between the facial and vocal modalities of expression for both the stock characters and basic emotions, extending previous work on verbal and non-verbal forms of expressive performance. These results not only provide new insight into the nature of acting and performance, but reveal the prospect of studying facial expression in the context of speech and dynamic performance, rather than producing static poses in isolation.

## Supplemental Material

sj-docx-1-qjp-10.1177_17470218211047935 – Supplemental material for The dynamic mask: Facial correlates of character portrayal in professional actors Supplemental material, sj-docx-1-qjp-10.1177_17470218211047935 for The dynamic mask: Facial correlates of character portrayal in professional actors by Matthew Berry and Steven Brown in Quarterly Journal of Experimental Psychology

sj-docx-2-qjp-10.1177_17470218211047935 – Supplemental material for The dynamic mask: Facial correlates of character portrayal in professional actors Supplemental material, sj-docx-2-qjp-10.1177_17470218211047935 for The dynamic mask: Facial correlates of character portrayal in professional actors by Matthew Berry and Steven Brown in Quarterly Journal of Experimental Psychology
