# Supplementary material for: The dynamic mask: Facial correlates of character portrayal in professional actors
Source: Q J Exp Psychol (Hove). 2021 Oct 4;75(5):936–53. doi: 10.1177/17470218211047935 (PMC8958566; doi:10.1177/17470218211047935)
Supplement: sj-docx-1-qjp-10.1177_17470218211047935 – Supplemental material for The dynamic mask: Facial correlates of character portrayal in professional actors [file sj-docx-1-qjp-10.1177_17470218211047935.docx]

Supplementary Material for:

**The dynamic mask: Facial correlates of character portrayal in professional actors**

Matthew Berry, Steven Brown


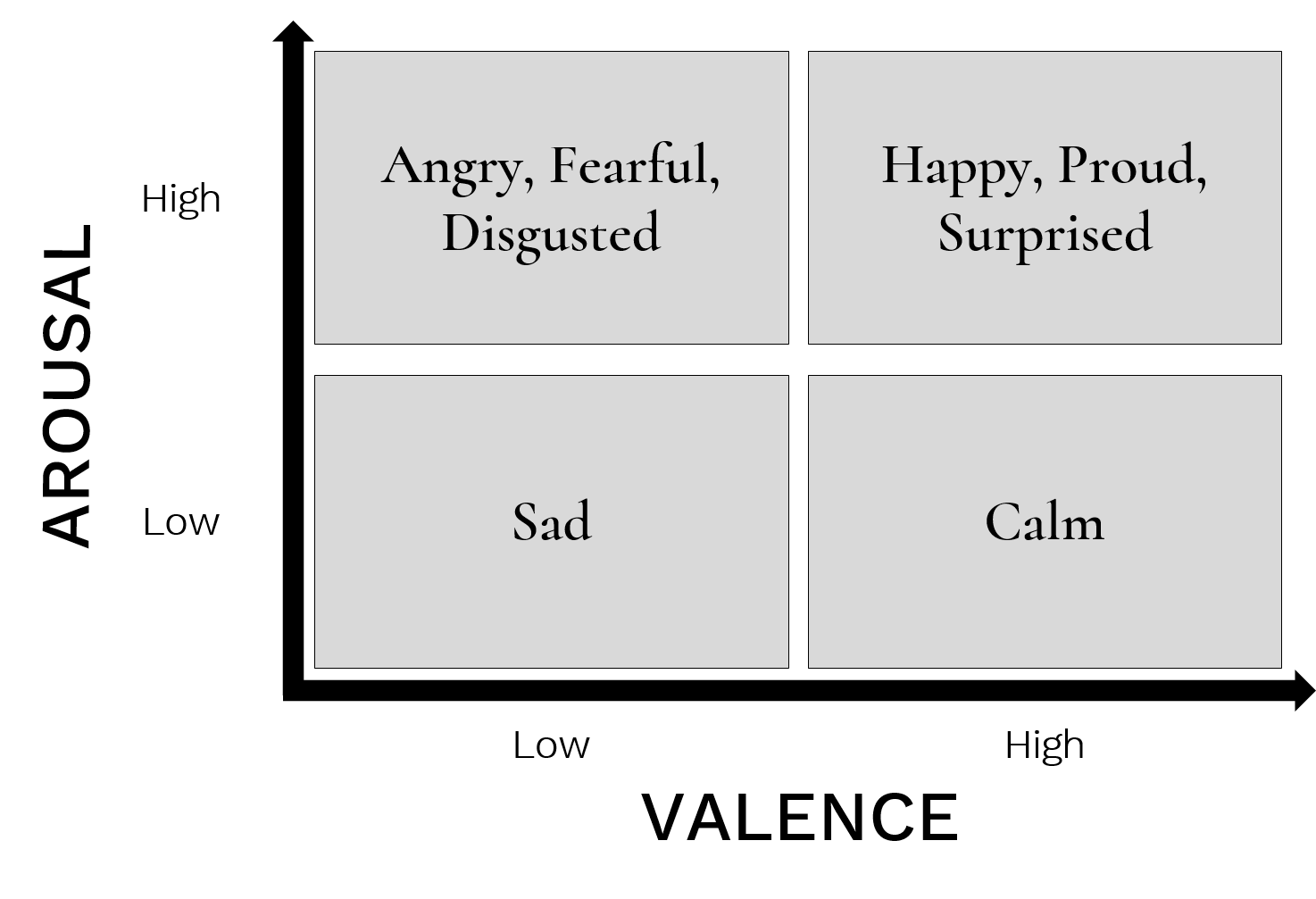
**Supplementary Figure 1. Emotion scheme*.*** The figure shows the 8 basic emotions used in the study, as organized into a 2 x 2 scheme based on a crossing of 2 levels of arousal and valence, respectively. This scheme approximates the circumplex model of emotions from Russell (1980).


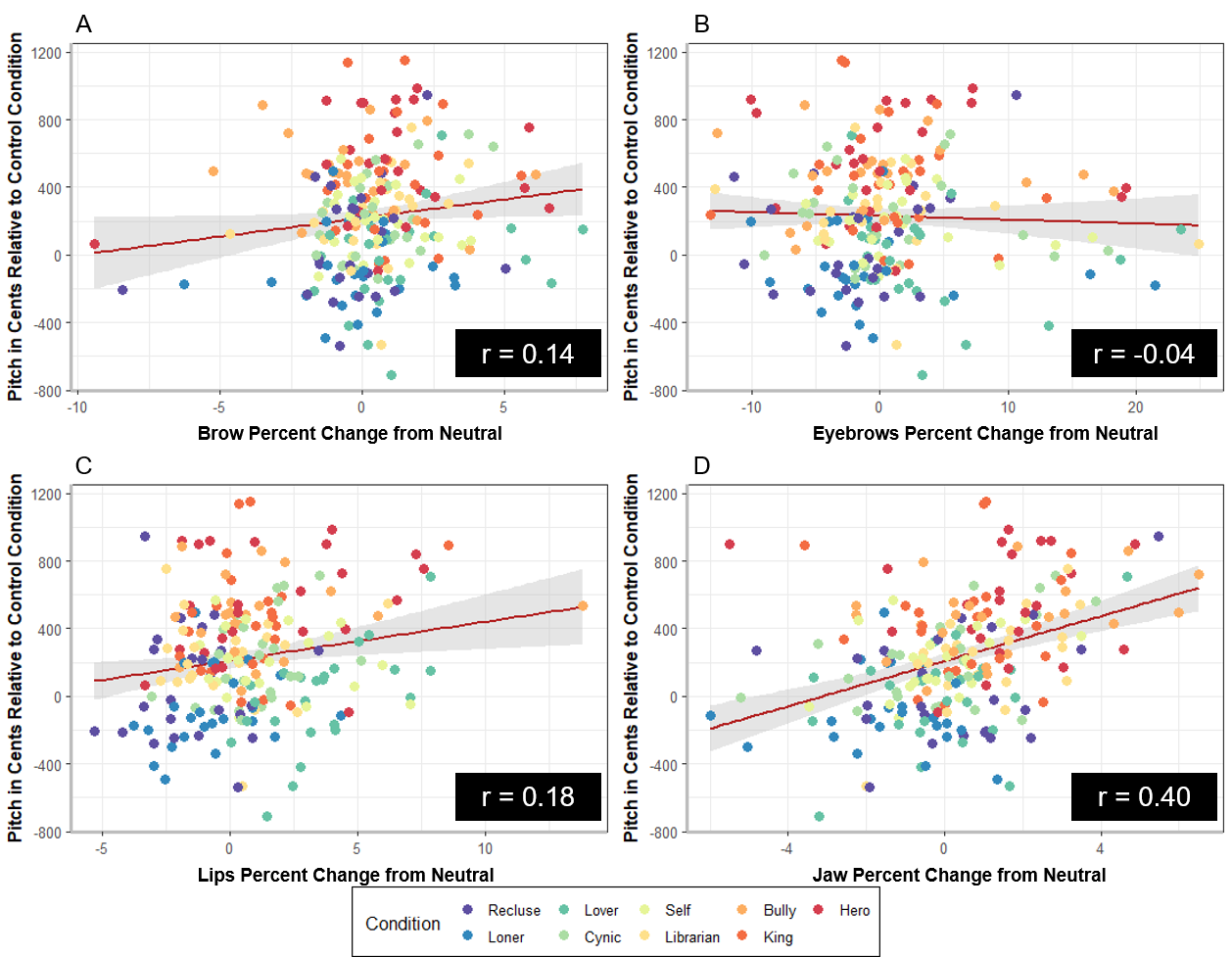


**Supplementary Figure 2. Character-condition linear regressions***.* (A) Character pitch production in cents relative to control condition as a function of brow expansion in percent change relative to the neutral emotion condition. (B) Character pitch production in cents relative to control condition as a function of eyebrow expansion in percent change relative to the neutral emotion condition. (C) Character pitch production in cents relative to control condition as a function of lip expansion in percent change relative to the neutral emotion condition. (D) Character pitch production in cents relative to control condition as a function of jaw expansion in percent change relative to the neutral emotion condition. Each point represents an observation from a single character-condition trial. The regression line is depicted in red. The standard error of the regression line is depicted in grey around the regression line. Pearson’s product-moment correlation r-values are depicted in black and are summarized in Table 2.


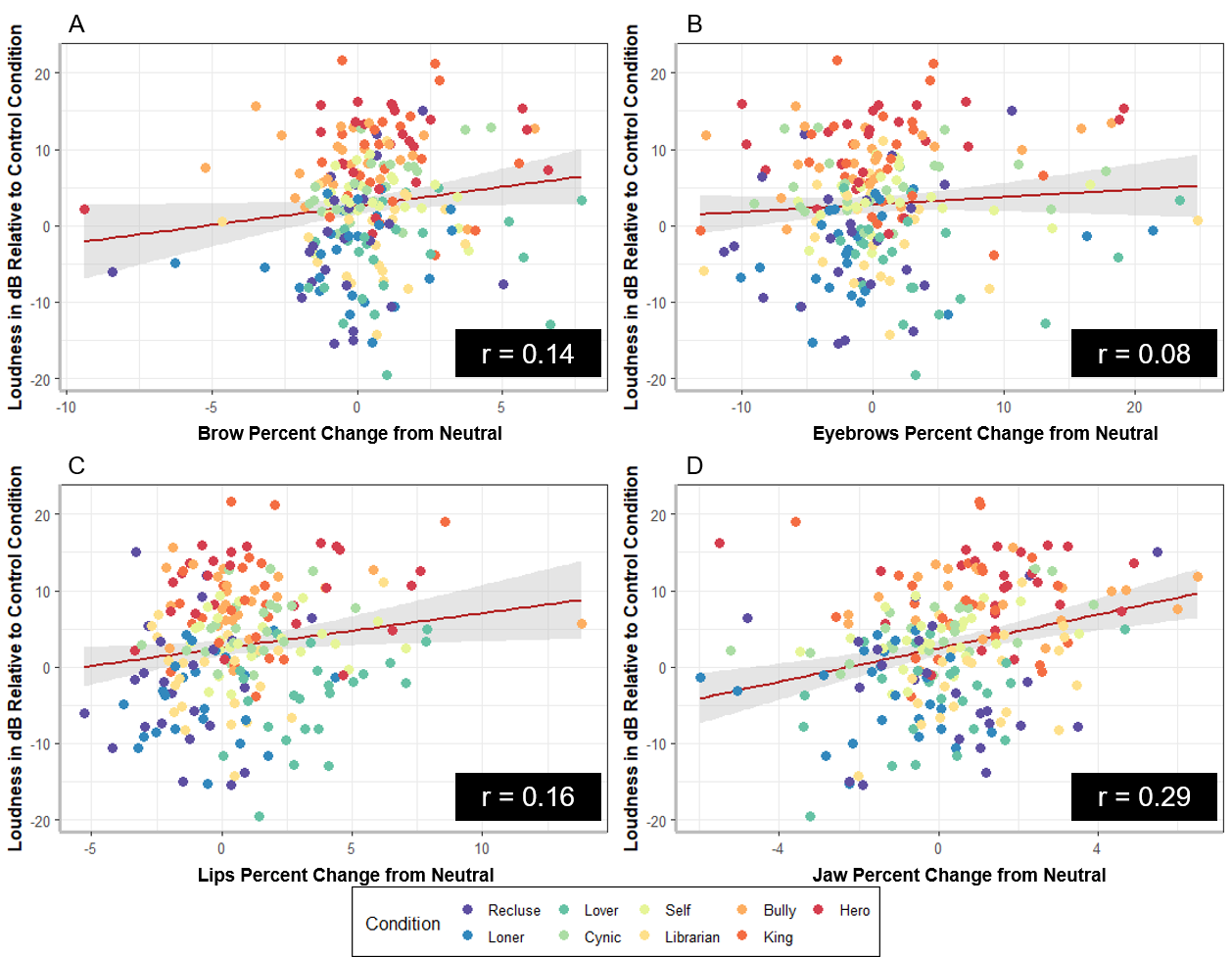


**Supplementary Figure 3. Character-condition linear regressions***.* (A) Character loudness production in decibels relative to control condition as a function of brow expansion in percent change relative to the neutral emotion condition. (B) Character loudness production in decibels relative to control condition as a function of eyebrow expansion in percent change relative to the neutral emotion condition. (C) Character loudness production in decibels relative to control condition as a function of lip expansion in percent change relative to the neutral emotion condition. (D) Character loudness production in decibels relative to control condition as a function of jaw expansion in percent change relative to the neutral emotion condition. Each point represents an observation from a single character-condition trial. The regression line is depicted in red. The standard error of the regression line is depicted in grey around the regression line. Pearson’s product-moment correlation r-values are depicted in black and are summarized in Table 2.


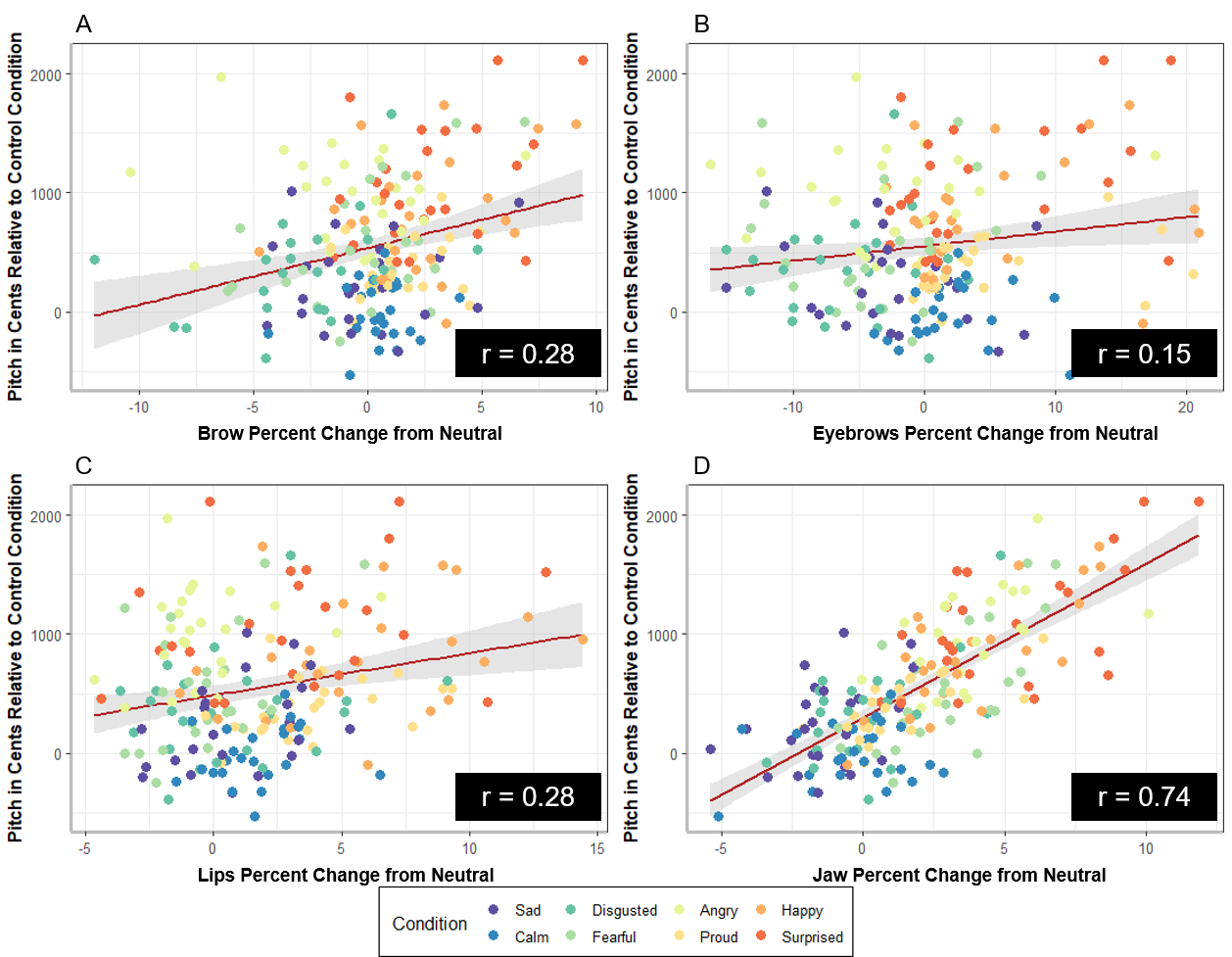


**Supplementary Figure 4. Emotion-condition linear regressions***.* (A) Emotion pitch production in cents relative to control condition as a function of brow expansion in percent change relative to the neutral emotion condition. (B) Emotion pitch production in cents relative to control condition as a function of eyebrow expansion in percent change relative to the neutral emotion condition. (C) Emotion pitch production in cents relative to control condition as a function of lip expansion in percent change relative to the neutral emotion condition. (D) Emotion pitch production in cents relative to control condition as a function of jaw expansion in percent change relative to the neutral emotion condition. Each point represents an observation from a single emotion-condition trial. The regression line is depicted in red. The standard error of the regression line is depicted in grey around the regression line. Pearson’s product-moment correlation r-values are depicted in black and are summarized in Table 2.


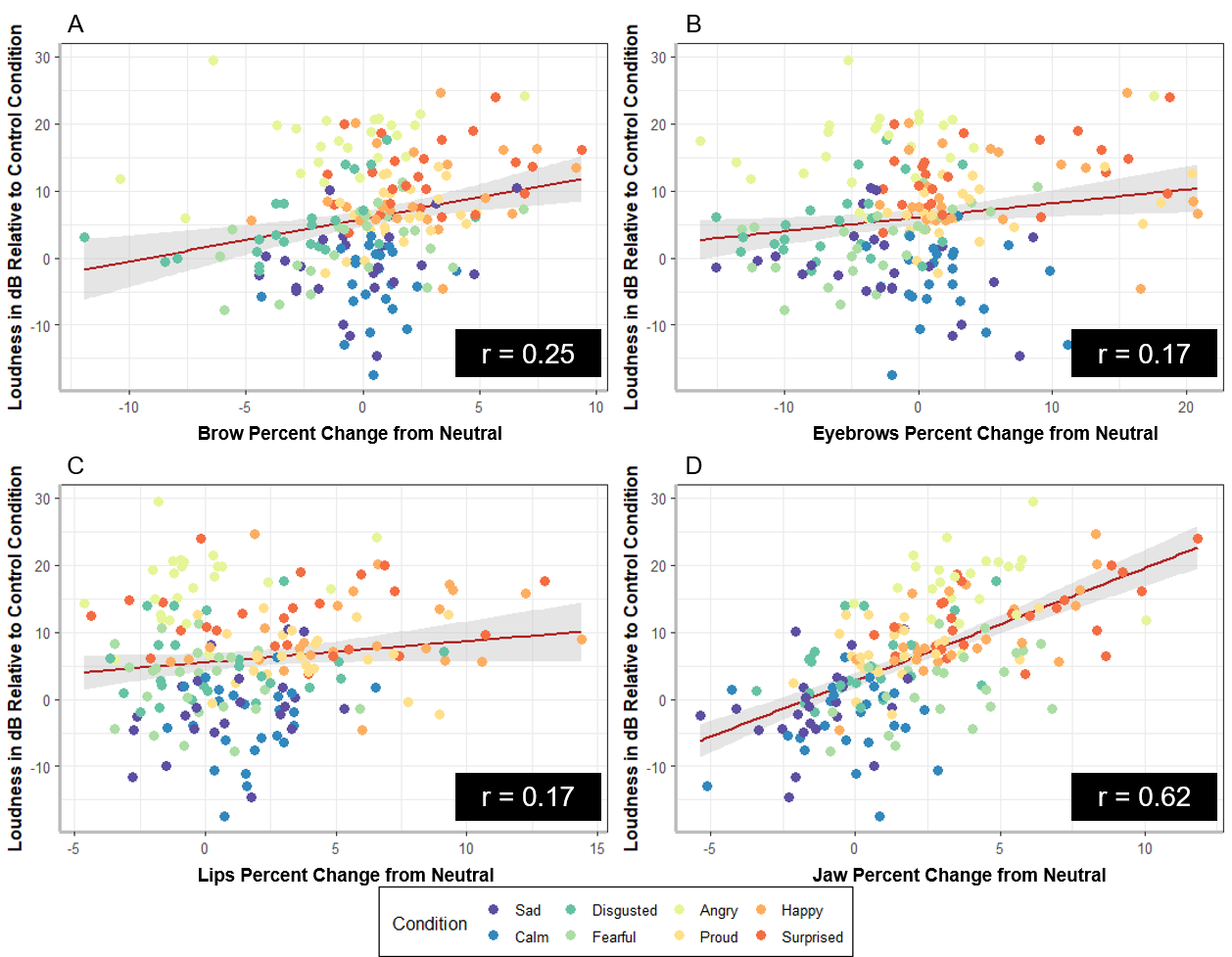


**Supplementary Figure 5. Emotion-condition linear regressions***.* (A) Emotion loudness production in decibels relative to control condition as a function of brow expansion in percent change relative to the neutral emotion condition. (B) Emotion loudness production in decibels relative to control condition as a function of eyebrow expansion in percent change relative to the neutral emotion condition. (C) Emotion loudness production in decibels relative to control condition as a function of lip expansion in percent change relative to the neutral emotion condition. (D) Emotion loudness production in decibels relative to control condition as a function of jaw expansion in percent change relative to the neutral emotion condition. Each point represents an observation from a single emotion-condition trial. The regression line is depicted in red. The standard error of the regression line is depicted in grey around the regression line. Pearson’s product-moment correlation r-values are depicted in black and are summarized in Table 2.

*Supplementary Table 1.* Emotion ANOVA table

| Segment | Direction | Effect Type | Sum Sq | Mean Sq | NumDF | DenDF | *F-value* | *p-value* | sig | *Rmarg^2^* | *Rcond^2^* |
| --- | --- | --- | --- | --- | --- | --- | --- | --- | --- | --- | --- |
| BROW | Vertical | Valence | 160.51 | 160.51 | 1 | 168 | 30.3 | 0.000 | *** | 0.22 | 0.50 |
|  |  | Arousal | 3.87 | 3.87 | 1 | 168 | 0.70 | 0.394 | n.s. |  |  |
|  |  | Valence x Arousal | 71.33 | 71.33 | 1 | 168 | 13.4 | 0.000 | *** |  |  |
| EYEBROWS | Horizontal | Valence | 1642.35 | 1642.35 | 1 | 168 | 90.5 | 0.000 | *** | 0.31 | 0.65 |
|  |  | Arousal | 15.06 | 15.06 | 1 | 168 | 0.80 | 0.364 | n.s. |  |  |
|  |  | Valence x Arousal | 153.43 | 153.43 | 1 | 168 | 8.5 | 0.004 | ** |  |  |
| LIPS | Horizontal | Valence | 230.91 | 230.91 | 1 | 168 | 37.5 | 0.000 | *** | 0.29 | 0.50 |
|  |  | Arousal | 22.75 | 22.75 | 1 | 168 | 3.70 | 0.056 | n.s. |  |  |
|  |  | Valence x Arousal | 106.02 | 106.02 | 1 | 168 | 17.2 | 0.000 | *** |  |  |
| JAW | Vertical | Valence | 39.68 | 39.68 | 1 | 168 | 7.40 | 0.007 | ** | 0.30 | 0.43 |
|  |  | Arousal | 475.07 | 475.07 | 1 | 168 | 88.6 | 0.000 | *** |  |  |
|  |  | Valence x Arousal | 0.00 | 0.00 | 1 | 168 | 0.00 | 0.994 | n.s. |  |  |
|  | | | | | | | | | | | |

*Note*: Summary of repeated measures analysis of variance (ANOVA) for each segment (relative to neutral, thereby controlling for speech). A linear mixed-effects regression analysis (LMER) was computed with subjects listed as the random effect and the two dimensions (Valence and Arousal) as the fixed effects. The ANOVA table includes type III sum of squares using Satterthwaite approximation for degrees of freedom. Measures of effect size indicate how much of the model's variance is explained by the fixed effects only (R_marg_^2^), and how much of the model's variance is explained by the complete (fixed + random effects) model (R_cond_^2^).* p < .05, **p < .01, ***p < .001. Abbreviations: SumSq (sum of squares), MeanSq (mean squares), NumDF (numerator degrees of freedom), DenDF (denominator degrees of freedom), sig (significance level), R_marg_^2^ (marginal r squared), R_cond_^2^ (conditional r squared).

*Supplementary Table 2.* Additional segments ANOVA table

| Condition | Segment | Side | Effect Type | Sum Sq | Mean Sq | NumDF | DenDF | *F-value* | *p-value* | sig | *Rmarg^2^* | *Rcond^2^* |
| --- | --- | --- | --- | --- | --- | --- | --- | --- | --- | --- | --- | --- |
| CHARACTERS | VERTICAL EYEBROWS | Left | Coop | 234.92 | 117.46 | 2 | 192 | 5.82 | 0.004 | ** | 0.07 | 0.44 |
|  |  |  | Assert | 131.24 | 65.62 | 2 | 192 | 3.25 | 0.041 | * |  |  |
|  |  |  | Coop x Assert | 170.49 | 42.62 | 4 | 192 | 2.11 | 0.081 | n.s |  |  |
|  |  | Right | Coop | 244.27 | 122.13 | 2 | 192 | 7.13 | 0.001 | ** | 0.08 | 0.48 |
|  |  |  | Assert | 121.39 | 60.69 | 2 | 192 | 3.54 | 0.031 | * |  |  |
|  |  |  | Coop x Assert | 174.13 | 43.53 | 4 | 192 | 2.54 | 0.041 | * |  |  |
|  | VERTICAL LIPS | Left | Coop | 5.95 | 2.98 | 2 | 192 | 3.13 | 0.046 | * | 0.20 | 0.43 |
|  |  |  | Assert | 11.28 | 5.64 | 2 | 192 | 5.93 | 0.003 | ** |  |  |
|  |  |  | Coop x Assert | 55.71 | 13.93 | 4 | 192 | 14.64 | 0.000 | *** |  |  |
|  |  | Right | Coop | 7.04 | 3.52 | 2 | 192 | 3.18 | 0.044 | * | 0.19 | 0.37 |
|  |  |  | Assert | 20.60 | 10.30 | 2 | 192 | 9.29 | 0.000 | *** |  |  |
|  |  |  | Coop x Assert | 43.47 | 10.87 | 4 | 192 | 9.81 | 0.000 | *** |  |  |
| EMOTIONS | VERTICAL EYEBROWS | Left | Valence | 1327.62 | 1327.62 | 1 | 168 | 43.9 | 0.000 | *** | 0.26 | 0.54 |
|  |  |  | Arousal | 11.03 | 11.03 | 1 | 168 | 0.40 | 0.547 | n.s. |  |  |
|  |  |  | Valence x Arousal | 366.56 | 366.56 | 1 | 168 | 12.1 | 0.001 | *** |  |  |
|  |  | Right | Valence | 1169.42 | 1169.42 | 1 | 168 | 39.0 | 0.000 | *** | 0.22 | 0.49 |
|  |  |  | Arousal | 22.48 | 22.48 | 1 | 168 | 0.70 | 0.388 | n.s. |  |  |
|  |  |  | Valence x Arousal | 206.97 | 206.97 | 1 | 168 | 6.90 | 0.009 | ** |  |  |
|  | VERTICAL LIPS | Left | Valence | 2.26 | 2.26 | 1 | 168 | 0.90 | 0.341 | n.s. | 0.01 | 0.30 |
|  |  |  | Arousal | 0.82 | 0.82 | 1 | 168 | 0.30 | 0.566 | n.s. |  |  |
|  |  |  | Valence x Arousal | 0.00 | 0.00 | 1 | 168 | 0.00 | 0.985 | n.s. |  |  |
|  |  | Right | Valence | 0.01 | 0.01 | 1 | 168 | 0.00 | 0.938 | n.s. | 0.01 | 0.14 |
|  |  |  | Arousal | 0.42 | 0.42 | 1 | 168 | 0.20 | 0.662 | n.s. |  |  |
|  |  |  | Valence x Arousal | 1.63 | 1.63 | 1 | 168 | 0.70 | 0.388 | n.s. |  |  |
|  |  |  |  |  |  |  |  |  |  |  |  |  |

*Note*: Summary of repeated measures analysis of variance (ANOVA) for each segment (relative to neutral, thereby controlling for speech). A linear mixed-effects regression analysis (LMER) was computed with subjects listed as the random effect and the two dimensions (Assertiveness and Cooperativeness for characters, Valence and Arousal for emotions) as the fixed effects. The ANOVA table includes type III sum of squares using Satterthwaite approximation for degrees of freedom. Measures of effect size indicate how much of the model's variance is explained by the fixed effects only (R_marg_^2^), and how much of the model's variance is explained by the complete (fixed + random effects) model (R_cond_^2^).* p < .05, **p < .01, ***p < .001. Abbreviations: SumSq (sum of squares), MeanSq (mean squares), NumDF (numerator degrees of freedom), DenDF (denominator degrees of freedom), sig (significance level), R_marg_^2^ (marginal r squared), R_cond_^2^ (conditional r squared), Assert (assertiveness), Coop (cooperativeness).

*Supplementary Table 3.* Control vs Performed Self Paired-sample t-tests

| Segment | | Group 1 | Group 2 | Estimate | DF | Low Conf. | High Conf. | *T-value* | *p-value* | sig | *Cohen's d* | Magnitude |
| --- | --- | --- | --- | --- | --- | --- | --- | --- | --- | --- | --- | --- |
| BROW | | Control | Self | 0.01 | 23 | -0.42 | 0.43 | 0.03 | 0.976 | n.s | 0.0 | negligible |
| EYEBROW | | Control | Self | -1.53 | 23 | -2.93 | -0.12 | -2.25 | 0.034 | * | -0.5 | small |
| LIPS | | Control | Self | -1.22 | 23 | -2.09 | -0.35 | -2.90 | 0.008 | ** | -0.6 | moderate |
| JAW | | Control | Self | -0.95 | 23 | -1.74 | -0.16 | -2.50 | 0.020 | * | -0.5 | moderate |
|  |  |  |  |  |  |  |  |  |  |  |  |  |

*Note*: For Cohen’s *d*, negligible effect = 0, small effect =.2, moderate effect = .5, large effect = .8. * *p* < .05, ** *p* < .01, *** *p* < .001

*Supplementary Table 4.* Varimax-rotated principal component loading matrix for characters and emotions.

| Modality | Variable | Parameter | Rotated Components | | Communalities | Uniqueness | Complexity |
| --- | --- | --- | --- | --- | --- | --- | --- |
|  |  |  | RC1 | RC2 |  |  |  |
| FACE | BROW | Mean |  | 0.62 | 0.38 | 0.62 | 1 |
|  | EYEBROWS | Mean |  | 0.59 | 0.36 | 0.64 | 1.1 |
|  | LIPS | Mean |  | 0.52 | 0.28 | 0.72 | 1.1 |
|  | JAW | Mean | 0.73 |  | 0.54 | 0.46 | 1.1 |
| VOICE | PITCH | Mean | 0.94 |  | 0.90 | 0.10 | 1 |
|  |  | Standard Deviation | 0.90 |  | 0.81 | 0.19 | 1 |
|  |  | Range | 0.89 |  | 0.80 | 0.20 | 1 |
|  | LOUDNESS | Mean | 0.79 | 0.35 | 0.75 | 0.25 | 1.4 |
|  |  | Standard Deviation | 0.67 |  | 0.45 | 0.55 | 1 |
|  |  | Range | 0.85 |  | 0.75 | 0.25 | 1.1 |
|  | DURATION | Pause Number |  | -0.51 | 0.29 | 0.71 | 1.3 |
|  |  | Signal Duration |  | -0.40 | 0.19 | 0.81 | 1.4 |
|  |  | Pause Duration | -0.39 |  | 0.26 | 0.74 | 1.9 |
|  | TIMBRE | Jitter |  | -0.69 | 0.51 | 0.49 | 1.2 |
|  |  | Shimmer | -0.39 | -0.58 | 0.49 | 0.51 | 1.7 |
|  |  | Noise to Harmonics Ratio |  | -0.63 | 0.51 | 0.49 | 1.5 |
|  | Sum of Square Loadings | | 5.37 | 2.92 |  |  |  |
|  | Proportion of Variance | | 0.34 | 0.18 |  |  |  |
|  | Cumulative Variance | | 0.34 | 0.52 |  |  |  |
|  | Mean Item Complexity | |  |  |  |  | 1.2 |

*Note:* Component loadings under 0.35 are omitted. Communalities are the proportion of variance in the parameter explained by the two RPCs. Uniqueness is the proportion of variance in the parameter not shared with other parameters. Complexity is the number of components needed to account for the observed variables.
