# Supplementary material for: The dynamic mask: Facial correlates of character portrayal in professional actors
Source: Q J Exp Psychol (Hove). 2021 Oct 4;75(5):936–53. doi: 10.1177/17470218211047935 (PMC8958566; doi:10.1177/17470218211047935)
Supplement: sj-docx-2-qjp-10.1177_17470218211047935 – Supplemental material for The dynamic mask: Facial correlates of character portrayal in professional actors [file sj-docx-2-qjp-10.1177_17470218211047935.docx]

Appendix A

*Neutral Script.* **“**I walked into the room. A bag is in the room. It sits on the cabinet beside a clock. Digital clocks are common. There are four drawers in the cabinet. I see a rug on the floor. It looks to be expensive.”
